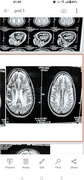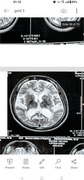# Chasing clues : Dementia unveils PML long after R‐CHOP treatment

**DOI:** 10.1002/alz70857_103459

**Published:** 2025-12-25

**Authors:** Swathi T, Thamil Pavai Swathi

**Affiliations:** ^1^ Kauvery hospital, Chennai, Tamil nadu, India; ^2^ Mmc, Chennai, Tamilnadu, India

## Abstract

**Background:**

PML is a very well known complication in immuno compromised patients, malignancy, and with disease modifying agents and chemotherapy agents. Here in we present a young male who had unsteadiness memory impairment, behavioral changes 3 year after being treated for NHL

**Method:**

33 year male treated for NHL of the spine 5 cycles RT A and chemotherapy R CHOP regimen 3 years back, presented with 10 months of intertwines, 4 months of memory impatient, dis inhibition, apathy, perseverance, with optic ataxia, occulomotor apraxia, finger agnosia, fab 5/18, cerebellar signs

**Result:**

A possibility of CNS Lymphoma, Paraneoplastic syndrome, PML, Prion was considered and evaluated. Mri brain shows white matter hyperintensity in subcortical, pv, cerebellar with involvement of u fibers, CSF normal and JC virus DNA In CSF positive

**Conclusion:**

Though there are case reports of R CHOP induced PML during 3 cycle or 4th, this case is rare by means of having PML afer 3 years of treatment highlighting, dementia presenting as PML unmasked.